# Incidence and outcomes of patients hospitalised with COPD in Greater Glasgow and Clyde, Scotland 2015–2022: a retrospective cohort study

**DOI:** 10.1186/s12890-026-04320-0

**Published:** 2026-05-26

**Authors:** Navpreet Kaur, Jim Lewsey, Christopher Carlin, Frederick Ho, Eve Walker, Claudia Geue, Daniel Mackay, Jocelyn Friday, Sandosh Padmanabhan, Jill P. Pell, Ruth Dundas, Tran Q. B. Tran, Denise Brown, Claire Hastie, Michael Fleming, Alan Stevenson, Clea du Toit

**Affiliations:** 1 School of Health and Wellbeing, Clarice Pears Building, 90 Byres Road, University of Glasgow, Glasgow, G12 8TB Scotland, UK; 2 School of Medicine, Dentistry & Nursing, University of Glasgow, Glasgow, G12 8TB Scotland, UK; 3 School of Cardiovascular and Metabolic Health, University of Glasgow, Glasgow, G12 8TB Scotland, UK; 4 Digital Health Validation Lab, Living Lab, University of Glasgow, Glasgow, G12 8TB Scotland, UK; 5 West of Scotland Innovation Hub, NHS Greater Glasgow and Clyde, Glasgow, Scotland, UK

**Keywords:** Chronic Obstructive Pulmonary Disease (COPD), Hospitalisation, Recurrent admissions, COVID-19, Scotland

## Abstract

**Background:**

Chronic Obstructive Pulmonary Disease (COPD) significantly impacts patients' quality of life and healthcare systems, especially among socio-economically deprived populations. Hospitalisations due to COPD exacerbations are critical events, often indicating declining health and higher mortality risk. This study examines the incidence and outcomes of COPD hospitalisations in National Health Service (NHS) Greater Glasgow and Clyde from 2015 to 2022, with a focus on socio-economic inequalities and the effects of the COVID-19 pandemic on hospitalisation trends.

**Methods:**

We conducted a retrospective cohort study using routinely collected hospital episode data, linked to mortality records, for a population of 1.2 million. Incident COPD hospitalisations were identified via International Classification of Diseases version 10 (ICD-10) codes, with no prior hospital admission in the preceding three years. Recurrent hospitalisations were those occurring within 3 years of the discharge of an incident event. We employed negative binomial regression to analyse hospital admission rates and Cox regression for time to recurrent hospitalisation and mortality outcomes, adjusting for demographic and comorbidity factors and testing for interactions with socio-economic status. Before getting the study sample, data linkage was done by the oGRE analyst team.

**Results:**

From 2015–2022, 8,852 incident COPD hospitalisations were recorded. The mean patient age was 70 years, and 61% were female. Incident hospitalisation rates declined by 13–48% from 2017 onwards. Mortality following incident hospital admission increased post-2020 (HR for 2022 vs. 2015 = 1.42, 95% CI 1.19–1.71). Recurrent hospitalisation rates declined significantly over the study period. Socio-economic inequalities were prominent, with the most deprived individuals experiencing higher hospitalisation and mortality rates.

**Conclusion:**

Our study highlights a reduction in COPD hospitalisations and recurrent admissions in recent years, potentially due to enhanced disease management strategies and pandemic-related healthcare changes. However, increased mortality post-hospitalisation and persistent socio-economic inequalities underscore the need for targeted interventions. Addressing these inequities through innovative care models and community-based support remains crucial to improving outcomes for patients with COPD.

**Supplementary Information:**

The online version contains supplementary material available at 10.1186/s12890-026-04320-0.

## Background

Chronic Obstructive Pulmonary Disease (COPD) is a common preventable and treatable disease which principally develops in patients over the age of 40, in association with cigarette smoking. COPD results in lung inflammation and damage, causing airflow obstruction, which leads to symptoms including breathlessness and exercise restriction, susceptibility to exacerbations, impairment of quality of life and adverse outcomes including hospitalisations, adverse cardiovascular events and premature mortality [1]. It is the 4th leading cause of death worldwide [2].

COPD is highly prevalent in populations, with incidence projected to rise substantially, compounded by a recognition that only a 1/3 of patients with the condition have a coded diagnosis. In Scotland, 134,000 people had a coded diagnosis of COPD in 2019, with a projected rise to 166,000 by 2044 [3]. COPD prevalence and adverse outcome rates, including hospitalisations and mortality, are highest in socio-economically deprived areas. In addition to the direct impact on patients with the condition, COPD places an unsustainable burden on healthcare systems and public finances [3]. Re-orientation of care, coupling early, accurate diagnosis with consistent adoption of proactive preventive interventions, presents the opportunity to reduce direct patient, service, and financial impacts of COPD [4]. A range of care transformation initiatives have been undertaken across organisations, and novel interventions which improve COPD outcomes are becoming available, with some successes with improved outcomes, clinical and cost-effectiveness, with the offset of inequalities notable [5–8].

COPD hospitalisations, especially for exacerbations, are pivotal events that often signify a decline in patient health and increase the risk of subsequent hospitalisations and death. COPD annual hospitalisation rates in Scotland declined by 48% in 2020 compared to the previous 5-year average, in association with the COVID-19 pandemic lockdown [9]. Previous studies have shown that cardiovascular-related risk factors were measured substantially less during COVID-19 pandemic lockdown and some have yet to recover by 2024, but there is no long term evaluation of COVID-19’s impact on COPD related outcomes [10]. There is concern that hospitalisation rates have relapsed, and that survival outcomes may have been adversely impacted, including by the interruptions to diagnostic services and routine preventative care consequent on pandemic-associated and subsequent healthcare delivery and capacity challenges. Understanding the pre- and post-pandemic patterns of COPD-related hospitalisations, including their incidence, recurrence, and associated survival outcomes stratified by socio-economic residential status, is a key requirement for prioritisation and planning of service delivery, pathway and care transformations, and to provide up to date reference routine clinical data at scale for comparisons when innovations are adopted. To address this in part, we have undertaken this evaluation using routine clinical data from the National Health Service (NHS) Greater Glasgow and Clyde, which supports a 1.2 m population, providing a microcosm for understanding COPD's impact in Scotland and offering insights that can inform wider healthcare strategies.

## Methods

### Study design

Retrospective cohort study; data available for identification of cohort and analysis between 1 st January 2012 and 31 st December 2022. We have used the RECORD checklist to guide our reporting (please see supplementary material-1).

### Setting and data sources

The cohort was identified from routinely collected hospital episode statistics for NHS Greater Glasgow and Clyde, the largest health board in Scotland, which provides services to 1.2 million people (approximately a quarter of the Scottish population). These statistics were linked to death records provided by the National Records of Scotland (NRS). Access to the dataset was within the trusted research environment platform provided by the West of Scotland Safe Haven. All unconsented data records are linked via a unique identification number (CHI number) before studying and research purposes. The CHI number of patients is updated regularly with health records, and the death records provided have in-depth insights about the cause and date of death. Before getting the study sample, data linkage was done by the oGRE analyst team.

### Identification of incident and recurrent COPD hospitalisations

A COPD hospitalisation was identified by an International Classification of Diseases version 10 (ICD-10) coding of J44 ‘Other chronic obstructive pulmonary disease’ and using all codes at levels below the J44 categorisation. An incident COPD hospitalisation was defined as a COPD hospitalisation in the primary diagnostic coding position with no preceding COPD hospitalisation in any of the 6 diagnostic coding positions, in the previous 3 years. A recurrent COPD hospitalisation was defined as a COPD hospitalisation occurring after discharge from the index COPD hospitalisation [11]. As sensitivity analyses, we used alternative definitions of incident and recurrent COPD hospitalisations, namely that the ICD-10 code could appear in any of the six diagnostic coding positions.

### Other variables

Covariates used in the statistical analysis include age at time of incident COPD hospitalisation, sex, Scottish Index of Multiple Deprivation (SIMD) decile, year of incident COPD hospitalisation and comorbidities identified from hospital records before the incident COPD hospitalisation of Cerebrovascular disease, Congestive Heart Failure, Dementia, Diabetes (organ), Diabetes (uncomplicated), Hemiplegia, Malignancy, Metastatic, Myocardial infarction, Mild liver disease, Severe liver disease, Peptic ulcer, Pulmonary disease, Peripheral Vascular Disease (PVD), Renal disease and Rheumatic disease.

### Statistical analysis

To calculate rates of incident COPD hospitalisation by age, sex, SIMD and year, we used whole population denominator data obtained from NRS. These rates were modelled using negative binomial regression. Time to death was modelled using Cox regression with all covariates. Survival times were censored at the end of 2022. Time to first recurrent COPD hospitalisation was modelled with all covariates using Cox regression with censoring occurring at the end of 2022 or at the time of death, i.e., using cause-specific hazard ratio for competing risk. Proportional hazards were assessed by visual inspection and testing of Schoenfeld residuals. For the modelling of time to death and time to first recurrent COPD hospitalisation, we used restricted cubic splines to assess whether age had a curvilinear association with the log-hazard of the outcome. We also modelled the rate of recurrent COPD hospitalisations with the count of future hospitalisations as the numerator and follow-up time as the denominator. These rates were calculated for a maximum follow-up period of 3 years due to our definition of incident COPD hospitalisation, allowing for the same individual to provide more than one incident case if the gap in time between admissions was at least 3 years. In all models, we tested for an interaction between SIMD and year to assess whether inequalities in their distribution changed over time. All models were adjusted for age, sex, SIMD, year of incident COPD hospitalisation and the included comorbidities. These were regarded as potential confounders. The included comorbidities could increase the risk of severe COPD (and thus the hospitalisation) as well as mortality and readmission outcomes. A significance level of 0.05 was used throughout, and analyses were conducted using Stata version 18.0.

### Ethics & data extraction methods

Delegated research ethics approval was granted for linkage to NHS patient data by the Local Privacy and Advisory Committee at NHS Greater Glasgow and Clyde. Cohorts and de-identified linked data were prepared by the West of Scotland Safe Haven Research Database at NHS Greater Glasgow and Clyde (IRAS Project ID 321198, REC reference 22/WS/1063).

### Data availability

Datasets used for specific West of Scotland Safe Haven projects may be made available on request, with appropriate ethical permissions and in accordance with standard Safe Haven security policies and procedures. Contact: West of Scotland Safe Haven team (ggc.safehaven.admin@nhs.scot).

## Results

### Demographics

In total, there were 8,852 incident COPD hospitalisations between 2015 and 2022. The mean age of individuals at the time of incident hospitalisation was 70 years, and approximately 61 in 100 were female (Table 1). Over half of the cohort reside in areas with the highest fifth of socioeconomic deprivation as measured by SIMD. The most common comorbidities were Pulmonary disease, Malignancy, Myocardial infarction and Diabetes.

**Table 1:** Demographics of 8,852 individuals hospitalised with an incident COPD hospitalisation

Variable	Number (%)
Age, years	69.8 (12.0)^*^, 70.6 (61.7, 78.7)^**^
Sex, male	3464 (39.1)
Year of hospitalisation	
2015	1361 (15.4)
2016	1465 (16.6)
2017	1210 (13.7)
2018	1060 (12.0)
2019	1238 (14.0)
2020	718 (8.1)
2021	776 (8.8)
2022	1024 (11.6)
Socio-economic deprivation, SIMD	
1 (most deprived)	3292 (37.2)
2	1733 (19.6)
3	910 (10.3)
4	627 (7.1)
5	510 (5.8)
6	446 (5.0)
7	381 (4.3)
8	284 (3.2)
9	324 (3.7)
10 (least deprived)	230 (2.6)
Missing	115 (1.3)
Comorbidities	
Cerebrovascular disease	890 (10.1)
Congestive Heart Failure	763 (8.6)
Dementia	321 (3.6)
Diabetes (organ)	61 (0.7)
Diabetes (uncomplicated)	1007 (11.4)
Hemiplegia	113 (1.3)
Malignancy	1326 (15.0)
Metastatic	243 (2.8)
Myocardial infarction	961 (10.9)
Mild liver disease	485 (5.5)
Severe liver disease	97 (1.1)
Peptic ulcer	507 (5.7)
Pulmonary disease	7476 (84.5)
Peripheral Vascular Disease (PVD)	732 (8.3)
Renal disease	745 (8.4)
Rheumatic disease	342 (3.9)

^*^Mean (SD)

^**^Median (interquartile range)

#### Rates of incident COPD hospitalisations

**Fig. 1:**
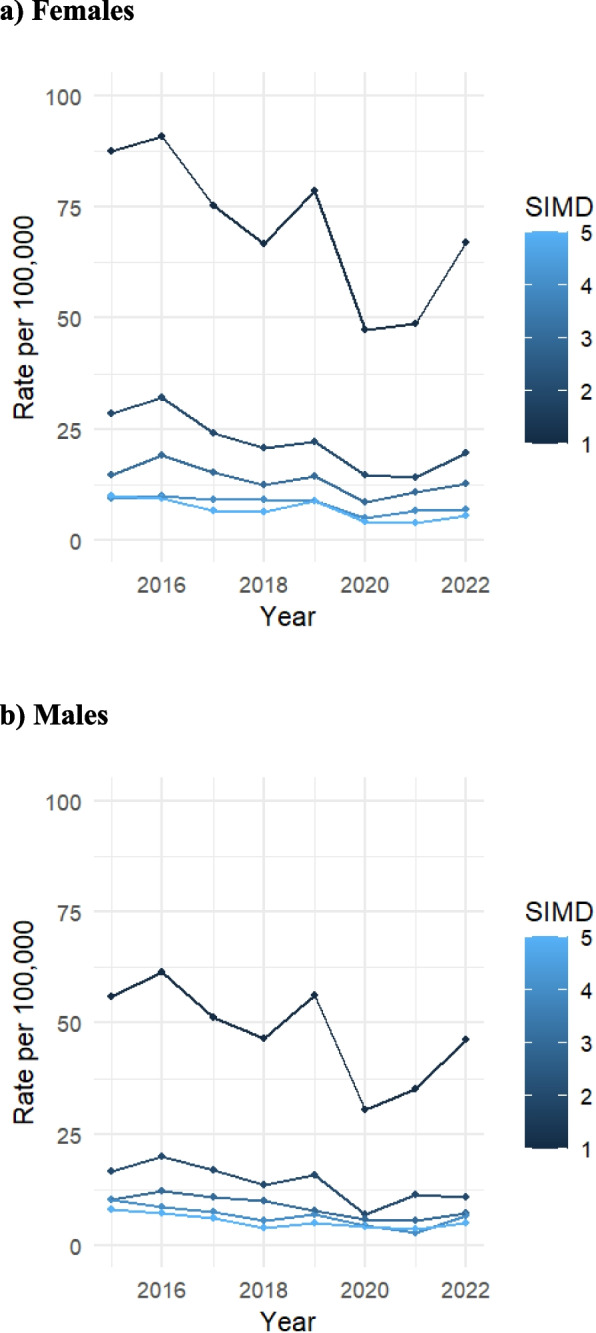
Crude incident COPD hospitalisation rates by year and socio-economic deprivation quintiles (1: most deprived, 5: least deprived) for **a**) Females and **b**) Males

The overall rate was 20.3 per 100,000 population (95% CI 19.9, 20.8) between 2015 and 2022. The sex-specific incident rates by year and socio-economic deprivation quintiles are shown in Fig. 1. Table 2 shows the rate ratios (RRs) for modelling incident COPD hospitalisations. For all years from 2017 onwards, there has been a 13–48% reduction in incident COPD hospitalisations compared to 2015. There is very strong socio-economic patterning to the rates of incident COPD hospitalisations (e.g., RR for SIMD 10 vs. 1 = 0.06 95% CI (0.05, 0.07)).

**Table 2:** Negative binomial regression results for modelling incident COPD hospitalisations

Variable	RR	95% CI		*p*-value
Age group, years				
60–69	9.09	8.37,	9.87	< 0.001
70–79	15.26	14.06,	16.56	< 0.001
80 +	19.44	17.74,	21.30	< 0.001
Sex, male	0.80	0.75,	0.84	< 0.001
Year of hospitalisation				
2016	1.08	0.98,	1.20	0.131
2017	0.87	0.78,	0.97	0.009
2018	0.74	0.67,	0.83	< 0.001
2019	0.85	0.77,	0.95	0.003
2020	0.52	0.46,	0.58	< 0.001
2021	0.55	0.49,	0.62	< 0.001
2022	0.69	0.62,	0.77	< 0.001
Socio-economic deprivation, SIMD				
2	0.47	0.43,	0.51	< 0.001
3	0.24	0.21,	0.26	< 0.001
4	0.16	0.14,	0.18	< 0.001
5	0.12	0.11,	0.14	< 0.001
6	0.11	0.10,	0.12	< 0.001
7	0.10	0.08,	0.11	< 0.001
8	0.07	0.06,	0.08	< 0.001
9	0.08	0.07,	0.09	< 0.001
10 (least deprived)	0.06	0.05,	0.07	< 0.001

The reference categories for age group, sex, year and SIMD are < 60 years, females, 2015 and decile 1, respectively

### Time to death

During follow-up, there were 3,825 deaths. Table 3 shows the hazard ratios (HRs) for modelling time to death. There was no evidence that age had a curvilinear association with log-hazard of time to death, so linearity was assumed and age fitted as a numerical covariate, and proportional hazards was a reasonable assumption for all covariates upon inspection and testing of Schoenfeld residuals. From 2020–2022 the risk of death following an incident COPD hospitalisation was higher than in 2015 (e.g., HR for 2022 vs. 2015 = 1.42 (1.19, 1.71). Those living in the least deprived areas had a lower risk of death than those living in the most deprived areas (HR for SIMD 10 vs. 1 = 0.61 95% CI (0.50, 0.76)).

**Table 3:** Cox regression results for time to death outcome

Variable	HR	95% CI		*p*-value
Age, years	1.06	1.05,	1.06	< 0.001
Sex, male	1.29	1.21,	1.38	< 0.001
Year of hospitalisation				
2016	1.00	0.91,	1.11	0.938
2017	0.99	0.89,	1.10	0.85
2018	1.09	0.97,	1.22	0.155
2019	1.02	0.91,	1.15	0.749
2020	1.11	0.96,	1.29	0.167
2021	1.28	1.09,	1.50	0.003
2022	1.42	1.19,	1.71	< 0.001
Socio-economic deprivation, SIMD				
2	0.99	0.91,	1.08	0.854
3	0.91	0.81,	1.01	0.088
4	0.95	0.83,	1.08	0.452
5	0.81	0.70,	0.93	0.004
6	0.98	0.85,	1.13	0.778
7	1.03	0.89,	1.19	0.682
8	0.99	0.83,	1.17	0.865
9	0.92	0.78,	1.08	0.307
10 (least deprived)	0.61	0.50,	0.76	< 0.001
Comorbidities				
Cerebrovascular disease	1.09	0.98,	1.20	0.098
Congestive Heart Failure	1.40	1.26,	1.56	< 0.001
Dementia	1.67	1.46,	1.92	< 0.001
Diabetes (organ)	0.89	0.62,	1.29	0.541
Diabetes (uncomplicated)	1.14	1.03,	1.25	0.011
Hemiplegia	1.17	0.88,	1.56	0.266
Malignancy	1.38	1.27,	1.51	< 0.001
Metastatic	2.24	1.89,	2.66	< 0.001
Myocardial infarction	0.99	0.90,	1.09	0.848
Mild liver disease	1.07	0.91,	1.25	0.428
Severe liver disease	1.58	1.18,	2.13	0.002
Peptic ulcer	1.21	1.06,	1.37	0.004
Pulmonary disease	1.09	0.99,	1.19	0.071
Peripheral Vascular Disease (PVD)	1.22	1.10,	1.36	< 0.001
Renal disease	1.10	0.99,	1.22	0.088
Rheumatic disease	1.08	0.93,	1.27	0.314

The reference categories for sex, year, SIMD and each comorbidity are females, 2015, decile 1, and no comorbidity present, respectively

### Time to first recurrent COPD hospitalisation

During follow-up, there were 4,987 first recurrent COPD hospitalisations. There was no evidence that age had a curvilinear association with log-hazard of time to first recurrent COPD hospitalisation so linearity was assumed and age was fitted as a numerical covariate, and proportional hazards assumption was met for all covariates. Table 4 shows the hazard ratios (HRs) for modelling time to first recurrent COPD hospitalisation. From 2017–2022 the risk of recurrent hospitalisation following an incident COPD hospitalisation was lower than in 2015 (e.g., HR for 2022 vs. 2015 = 0.88 (0.76, 1.02). Those living in the least deprived areas had lower risk of recurrent hospitalisation than those living in the most deprived areas (HR for SIMD 10 vs. 1 = 0.71 95% CI (0.59, 0.86)).

**Table 4:** Cox regression results for time to first recurrent COPD hospitalisation outcome

Variable	HR	95% CI		*p*-value
Age, years	1.02	1.02,	1.02	< 0.001
Sex, male	1.08	1.02,	1.14	0.01
Year of hospitalisation				
2016	0.96	0.88,	1.05	0.391
2017	0.89	0.81,	0.98	0.017
2018	0.84	0.76,	0.93	0.001
2019	0.74	0.67,	0.82	< 0.001
2020	0.83	0.74,	0.94	0.003
2021	0.97	0.86,	1.10	0.67
2022	0.88	0.76,	1.02	0.08
Socio-economic deprivation, SIMD				
2	1.00	0.92,	1.08	0.939
3	0.98	0.89,	1.08	0.661
4	0.96	0.86,	1.08	0.506
5	0.93	0.83,	1.06	0.285
6	0.95	0.83,	1.09	0.462
7	0.98	0.85,	1.12	0.73
8	0.85	0.72,	1.01	0.065
9	0.79	0.68,	0.93	0.005
10 (least deprived)	0.71	0.59,	0.86	< 0.001
Comorbidities				
Cerebrovascular disease	1.00	0.91,	1.11	0.929
Congestive Heart Failure	1.28	1.15,	1.42	< 0.001
Dementia	0.87	0.74,	1.03	0.11
Diabetes (organ)	0.87	0.61,	1.24	0.432
Diabetes (uncomplicated)	1.11	1.02,	1.22	0.02
Hemiplegia	1.43	1.13,	1.81	0.003
Malignancy	1.08	0.99,	1.18	0.068
Metastatic	1.12	0.92,	1.38	0.255
Myocardial infarction	1.08	0.99,	1.18	0.096
Mild liver disease	1.25	1.10,	1.42	0.001
Severe liver disease	0.83	0.61,	1.12	0.219
Peptic ulcer	1.15	1.02,	1.29	0.02
Pulmonary disease	1.15	1.07,	1.25	< 0.001
Peripheral Vascular Disease (PVD)	1.24	1.13,	1.37	< 0.001
Renal disease	0.88	0.79,	0.98	0.024
Rheumatic disease	0.89	0.77,	1.04	0.147

The reference categories for sex, year, SIMD and each comorbidity are females, 2015, decile 1, and no comorbidity present, respectively

### Rates of recurrent COPD hospitalisations

For all years from 2017 onwards, there has been a strong trend in reduction of rates for recurrent COPD hospitalisations compared to 2015 (e.g., RR for 2022 vs. 2015 = 0.20 95% CI (0.16, 0.26)) see Table 5. There is very strong socio-economic patterning to the rates of recurrent COPD hospitalisations (e.g., RR for SIMD 10 vs. 1 = 0.63 95% CI (0.42, 0.95)).

**Table 5:** Negative binomial regression results for modelling rates of recurrent COPD hospitalisations

Variable	RR	95% CI		*p*-value
Age group, years				
60–69	0.90	0.75,	1.07	0.234
70–79	1.30	1.09,	1.55	0.004
80 +	1.09	0.90,	1.34	0.378
Sex, male	1.28	1.12,	1.45	< 0.001
Year of hospitalisation				
2016	0.90	0.73,	1.10	0.302
2017	1.06	0.85,	1.33	0.618
2018	0.90	0.72,	1.14	0.383
2019	0.61	0.49,	0.76	< 0.001
2020	0.38	0.29,	0.50	< 0.001
2021	0.60	0.46,	0.78	< 0.001
2022	0.20	0.16,	0.26	< 0.001
Socio-economic deprivation, SIMD				
2	0.94	0.79,	1.11	0.474
3	0.88	0.70,	1.10	0.248
4	0.96	0.75,	1.23	0.727
5	0.95	0.72,	1.25	0.707
6	1.16	0.86,	1.57	0.335
7	0.76	0.55,	1.05	0.091
8	0.62	0.44,	0.90	0.011
9	0.63	0.45,	0.89	0.009
10 (least deprived)	0.63	0.42,	0.95	0.027

The reference categories for age group, sex, year and SIMD are < 60 years, females, 2015 and decile 1, respectively

### Inequalities

None of the testing of interactions between SIMD and year was statistically significant.

### Sensitivity analyses

The results were similar when using all six diagnostic positions to define COPD hospitalisation [see supplementary material-2, Additional Tables A1-A5]. When COPD hospitalisation was in a secondary position, the most common primary coded diagnoses were of Pneumonia, unspecified (J189), with 1,282 cases (5.38%), followed by Lobar Pneumonia, unspecified (J181) with 923 cases (3.88%), atherosclerotic heart disease (I251) with 707 cases (2.97%), and chest pain, unspecified (R074) with 647 cases (2.72%).

## Discussion

This study provides a comprehensive evaluation of the temporal changes in COPD hospitalisation and outcomes in Scotland’s most populous health board for the period 2015–2022, spanning the COVID-19 pandemic. Using routinely collected episode data from a population of 1.2 million linked to mortality records, our findings detail the dual impact of socio-economic inequalities and the COVID-19 pandemic on COPD-related hospitalisation trends. Using the main diagnostic position for identifying COPD has been shown to be highly accurate in Scotland [12]. Our analyses indicate a decline in the incidence of COPD-related hospitalisation before the COVID-19 pandemic (2017–2019), with stable survival rates and a decrease in the time to both readmission and the rate of readmission. The dataset importantly demonstrates the impact of inequalities, with higher COPD hospitalisation and mortality rates among the socio-economically deprived population.

During the COVID-19 pandemic (2020–2022), the incident COPD hospitalisation rates further decreased in part due to pandemic-related service disruption, along with a rise in the time-to-death rate following COPD-related admission, while observing a downward trend in the readmission and time-to-readmission rates following a COPD-related admission. There was a notable reduction in the rate of incident COPD hospitalisations in 2020, and this cannot be related to a reduction in COPD prevalence in the population, which has been confirmed to be rising in Scotland [3]. However, in 2021 and 2022, COPD hospitalisation rates increased and mortality following an incident COPD hospitalisation increased from 2020 to 2022 compared to 2015. This highlights a complex interplay between hospitalisation rates and survival outcomes, with potential influences including positive impact of hospital-avoidance care interventions including pandemic-related shielding with reduced exposure to pollution and infections, but negative impacts from higher acuity at hospitalisation and/or poorer functional status and physiological reserve at hospitalisation.

The temporal changes observed from 2020 to 2022 were likely due to the impact of the COVID-19 pandemic on overall hospitalisation rates and the interruption of routine care. While the survival outcomes were impaired compared to previous years, readmission rates remained stable, which could be attributed to the positive or parallel effects of care transformation initiatives in Greater Glasgow & Clyde, such as Community Respiratory Response Teams (CRRT) and related services [13]. Additionally, throughout the study period, we observed a consistent correlation between socioeconomic inequality and COPD incident hospitalisation, readmission and overall survival outcomes.

The rate of recurrent COPD hospitalisations showed a strong declining trend from 2019 onwards. This decline could reflect improvements in post-discharge care and disease management strategies aimed at preventing exacerbations and readmissions. However, socio-economic inequalities in hospitalisation rates persist, with patients from the most deprived areas continuing to experience higher rates of both incident and recurrent hospitalisations.

The socio-economic patterning of COPD hospitalisations and outcomes observed in our study aligns with broader health inequality trends. Those residing in the least deprived areas consistently exhibited better outcomes, including lower mortality and lower rates of recurrent hospitalisations, with this inequality increasing over time. Our findings are in line with previous studies from the UK and South Korea, which also highlighted increased smoking rates, higher rates of residence in cold and damp housing and increased exposure to poor quality air as partial contributors to the adverse outcomes experienced with deprivation [14]. Our findings highlight the critical need for targeted interventions to address the underlying determinants of health that contribute to these inequalities. Recent data from our group confirms that accessibility of care, and outcomes from that care for patients from deprived areas can be normalised with digitally-enabled pathway transformation, facilitating multi-disciplinary team delivery of evidence-based care interventions [7].

Significant efforts are underway within NHS Greater Glasgow and Clyde and across Scotland to transform COPD care and address these challenges. Initiatives such as the integration of community-based services, enhanced support for smoking cessation, and the implementation of personalised care plans are aimed at reducing hospital admissions and improving patient outcomes [10].

The temporal trends may in part reflect an overall improvement in care quality, which is likely attributed to positive care transformation interventions, such as cardiopulmonary risk management, community respiratory teams, home-based non-invasive ventilation services, and COPD digital health services. For instance, the COVID-19 pandemic has accelerated the adoption of telehealth and remote monitoring technologies, providing new avenues for managing chronic conditions like COPD outside traditional healthcare settings [7, 15, 16]. These innovations hold promise for further reducing hospitalisations and addressing inequalities in care, though their long-term impact remains to be fully assessed.

### Limitations

The study identifies several limitations. Comorbidities were assumed to act as confounders; however, the impact of comorbidities may also been influenced by the secular trends of COPD outcomes, and other residual confounding is likely in this observational study that uses routine hospital episode data which was not primarily collected for undertaking epidemiological research. For example, people might have become less sick over time, making them less susceptible to the outcome. Further, the small number of events reported in 2022 limits statistical precision and misclassification when using routine healthcare records is always a possibility, although accuracy is likely to be high in our data [12]. Finally, since the study's time frame ended in 2022, it only captures a single winter season (when COPD exacerbations and admissions are more prevalent), unaffected by COVID-19 and does not include an assessment of more recent trends.

## Conclusion

This study contributes to our understanding of the incidence and outcomes of COPD hospitalisations within a large and diverse population. Our findings highlight the ongoing challenges in managing COPD, particularly the persistent socio-economic inequalities in hospitalisation rates and outcomes. Addressing these inequities through targeted interventions and continuing to innovate in COPD care will be crucial for improving the lives of patients with this debilitating condition.

## Supplementary Information

Supplementary Material 1.

Supplementary Material 2.

## Data Availability

Datasets used for specific West of Scotland Safe Haven projects may be made available on request, with appropriate ethical permissions and in accordance with standard Safe Haven security policies and procedures. Contact: West of Scotland Safe Haven team (ggc.safehaven.admin@nhs.scot).

## Abbreviations

COPD: Chronic Obstructive Pulmonary Disease
NHS: National Health Service
NRS: National Records of Scotland
ICD-10: International Classification of Diseases version 10
SIMD: Scottish Index of Multiple Deprivation
PVD: Peripheral Vascular Disease
RRs: Rate Ratios
CRRT: Community Respiratory Response Teams
