# Supplementary material for: Incidence and outcomes of patients hospitalised with COPD in Greater Glasgow and Clyde, Scotland 2015–2022: a retrospective cohort study
Source: BMC Pulm Med. 2026 May 26;26:330. doi: 10.1186/s12890-026-04320-0 (PMC13403885; doi:10.1186/s12890-026-04320-0)
Supplement: Supplementary file 2 — Supplementary Material 2. [file 12890_2026_4320_MOESM2_ESM.docx]

**Supplementary materials**

**Additional Tables**

**Table A1:** Demographics of individuals hospitalised with an incident COPD hospitalisation

| **Variable** | **Number (%)** |
| --- | --- |
| Age, years | 70.8 (12.0)* |
| Sex, male | 14154 (43.3) |
| Year of hospitalisation |  |
| 2015 | 4500 (13.8) |
| 2016 | 4721 (14.5) |
| 2017 | 4154 (12.7) |
| 2018 | 3606 (11.0) |
| 2019 | 4149 (12.7) |
| 2020 | 3552 (10.9) |
| 2021 | 3988 (12.1) |
| 2022 | 3992 (12.2) |
| Socio-economic deprivation, SIMD |  |
| 1 (most deprived) | 11571 (35.4) |
| 2 | 6396 (19.6) |
| 3 | 3434 (10.5) |
| 4 | 2483 (7.6) |
| 5 | 1954 (6.0) |
| 6 | 1575 (4.8) |
| 7 | 1387 (4.3) |
| 8 | 1196 (3.7) |
| 9 | 1354 (4.2) |
| 10 (least deprived) | 945 (2.9) |
| Missing | 367 (1.1) |
| Comorbidities |  |
| Cerebrovascular disease | 4485 (13.7) |
| Congestive Heart Failure | 3469 (10.6) |
| Dementia | 1474 (4.5) |
| Diabetes (organ) | 368 (1.1) |
| Diabetes (uncomplicated) | 4624 (14.2) |
| Hemiplegia | 478 (1.5) |
| Malignancy | 6646 (20.4) |
| Metastatic | 1579 (4.8) |
| Myocardial infarction | 4893 (15.0) |
| Mild liver disease | 2170 (6.6) |
| Severe liver disease | 590 (1.8) |
| Peptic ulcer | 1964 (6.0) |
| Pulmonary disease | 27014 (82.7) |
| PVD | 3338 (10.2) |
| Renal disease | 3209 (9.8) |
| Rheumatic disease | 1275 (3.9) |
| * mean (SD) | |

**Table A2:** Negative binomial regression results for modelling incident COPD hospitalisations

| **Variable** | **RR** | **95% CI** | | **p-value** |
| --- | --- | --- | --- | --- |
| Age group, years |  |  |  |  |
| 60-69 | 10.19 | 9.55, | 10.88 | <0.001 |
| 70-79 | 19.44 | 18.22, | 20.75 | <0.001 |
| 80+ | 30.36 | 28.36, | 32.51 | <0.001 |
| Sex, male | 0.96 | 0.92, | 1.00 | 0.051 |
| Year of hospitalisation |  |  |  |  |
| 2016 | 1.02 | 0.94, | 1.12 | 0.576 |
| 2017 | 0.88 | 0.81, | 0.96 | 0.004 |
| 2018 | 0.74 | 0.68, | 0.81 | <0.001 |
| 2019 | 0.83 | 0.76, | 0.91 | <0.001 |
| 2020 | 0.71 | 0.65, | 0.78 | <0.001 |
| 2021 | 0.79 | 0.73, | 0.87 | <0.001 |
| 2022 | 0.79 | 0.72, | 0.86 | <0.001 |
| Socio-economic deprivation, SIMD |  |  |  |  |
| 2 | 0.49 | 0.45, | 0.54 | <0.001 |
| 3 | 0.25 | 0.23, | 0.27 | <0.001 |
| 4 | 0.17 | 0.16, | 0.19 | <0.001 |
| 5 | 0.13 | 0.12, | 0.14 | <0.001 |
| 6 | 0.10 | 0.09, | 0.11 | <0.001 |
| 7 | 0.09 | 0.08, | 0.09 | <0.001 |
| 8 | 0.07 | 0.07, | 0.08 | <0.001 |
| 9 | 0.08 | 0.08, | 0.09 | <0.001 |
| 10 (least deprived) | 0.06 | 0.05, | 0.06 | <0.001 |

**Table A3:** Cox regression results for time to death outcome

| **Variable** | **HR** | **95% CI** | | **p-value** |
| --- | --- | --- | --- | --- |
| Age, years | 1.05 | 1.05, | 1.05 | <0.001 |
| Sex, male | 1.23 | 1.19, | 1.27 | <0.001 |
| Year of hospitalisation |  |  |  |  |
| 2016 | 1.03 | 0.98, | 1.09 | 0.242 |
| 2017 | 1.05 | 0.99, | 1.11 | 0.105 |
| 2018 | 1.08 | 1.01, | 1.14 | 0.019 |
| 2019 | 1.04 | 0.98, | 1.11 | 0.19 |
| 2020 | 1.13 | 1.06, | 1.21 | <0.001 |
| 2021 | 1.10 | 1.02, | 1.18 | 0.015 |
| 2022 | 1.23 | 1.13, | 1.34 | <0.001 |
| Socio-economic deprivation, SIMD |  |  |  |  |
| 2 | 0.98 | 0.94, | 1.03 | 0.441 |
| 3 | 0.92 | 0.87, | 0.98 | 0.005 |
| 4 | 0.99 | 0.93, | 1.06 | 0.799 |
| 5 | 0.90 | 0.84, | 0.97 | 0.003 |
| 6 | 0.95 | 0.88, | 1.02 | 0.179 |
| 7 | 0.93 | 0.86, | 1.00 | 0.064 |
| 8 | 0.93 | 0.85, | 1.01 | 0.094 |
| 9 | 0.88 | 0.81, | 0.95 | 0.002 |
| 10 (least deprived) | 0.76 | 0.68, | 0.83 | <0.001 |
| Comorbidities |  |  |  |  |
| Cerebrovascular disease | 1.13 | 1.08, | 1.18 | <0.001 |
| Congestive Heart Failure | 1.42 | 1.35, | 1.49 | <0.001 |
| Dementia | 1.68 | 1.58, | 1.79 | <0.001 |
| Diabetes (organ) | 1.28 | 1.11, | 1.47 | 0.001 |
| Diabetes (uncomplicated) | 1.08 | 1.03, | 1.13 | 0.001 |
| Hemiplegia | 1.10 | 0.97, | 1.26 | 0.144 |
| Malignancy | 1.56 | 1.49, | 1.62 | <0.001 |
| Metastatic | 2.80 | 2.62, | 2.99 | <0.001 |
| Myocardial infarction | 0.93 | 0.89, | 0.97 | 0.001 |
| Mild liver disease | 1.16 | 1.07, | 1.25 | <0.001 |
| Severe liver disease | 1.78 | 1.58, | 2.01 | <0.001 |
| Peptic ulcer | 1.08 | 1.01, | 1.15 | 0.03 |
| Pulmonary disease | 1.10 | 1.05, | 1.15 | <0.001 |
| Peripheral Vascular Disease (PVD) | 1.19 | 1.13, | 1.25 | <0.001 |
| Renal disease | 1.12 | 1.06, | 1.17 | <0.001 |
| Rheumatic disease | 1.03 | 0.95, | 1.12 | 0.476 |

**Table A4:** Cox regression results for time to first recurrent COPD hospitalisation outcome

| **Variable** | **HR** | **95% CI** | | **p-value** |
| --- | --- | --- | --- | --- |
| Age, years | 1.02 | 1.02, | 1.02 | <0.001 |
| Sex, male | 1.04 | 1.00, | 1.07 | 0.035 |
| Year of hospitalisation |  |  |  |  |
| 2016 | 1.00 | 0.94, | 1.05 | 0.871 |
| 2017 | 0.90 | 0.85, | 0.95 | <0.001 |
| 2018 | 0.89 | 0.84, | 0.95 | <0.001 |
| 2019 | 0.86 | 0.81, | 0.91 | <0.001 |
| 2020 | 0.86 | 0.81, | 0.92 | <0.001 |
| 2021 | 0.86 | 0.81, | 0.92 | <0.001 |
| 2022 | 0.81 | 0.75, | 0.89 | <0.001 |
| Socio-economic deprivation, SIMD |  |  |  |  |
| 2 | 0.98 | 0.93, | 1.02 | 0.31 |
| 3 | 0.98 | 0.92, | 1.04 | 0.462 |
| 4 | 1.00 | 0.93, | 1.06 | 0.944 |
| 5 | 0.96 | 0.89, | 1.03 | 0.281 |
| 6 | 0.95 | 0.88, | 1.03 | 0.234 |
| 7 | 1.00 | 0.92, | 1.08 | 0.926 |
| 8 | 0.84 | 0.76, | 0.92 | <0.001 |
| 9 | 0.88 | 0.81, | 0.96 | 0.004 |
| 10 (least deprived) | 0.74 | 0.66, | 0.82 | <0.001 |
| Comorbidities |  |  |  |  |
| Cerebrovascular disease | 1.01 | 0.96, | 1.06 | 0.711 |
| Congestive Heart Failure | 1.13 | 1.07, | 1.19 | <0.001 |
| Dementia | 0.95 | 0.87, | 1.04 | 0.268 |
| Diabetes (organ) | 1.20 | 1.03, | 1.39 | 0.022 |
| Diabetes (uncomplicated) | 1.04 | 0.99, | 1.10 | 0.079 |
| Hemiplegia | 1.18 | 1.04, | 1.35 | 0.013 |
| Malignancy | 1.13 | 1.08, | 1.18 | <0.001 |
| Metastatic | 1.37 | 1.25, | 1.50 | <0.001 |
| Myocardial infarction | 1.08 | 1.03, | 1.13 | 0.001 |
| Mild liver disease | 1.18 | 1.10, | 1.27 | <0.001 |
| Severe liver disease | 1.15 | 1.01, | 1.31 | 0.037 |
| Peptic ulcer | 1.14 | 1.06, | 1.21 | <0.001 |
| Pulmonary disease | 1.16 | 1.11, | 1.22 | <0.001 |
| Peripheral Vascular Disease (PVD) | 1.15 | 1.09, | 1.21 | <0.001 |
| Renal disease | 1.02 | 0.96, | 1.08 | 0.529 |
| Rheumatic disease | 1.02 | 0.94, | 1.11 | 0.664 |

**Table A5:** Negative binomial regression results for modelling rates of recurrent COPD hospitalisations

| **Variable** | **RR** | **95% CI** | | **p-value** |
| --- | --- | --- | --- | --- |
| Age group, years |  |  |  |  |
| 60-69 | 0.90 | 0.80, | 1.00 | 0.05 |
| 70-79 | 1.12 | 1.01, | 1.25 | 0.04 |
| 80+ | 0.93 | 0.83, | 1.05 | 0.227 |
| Sex, male | 1.07 | 0.99, | 1.15 | 0.074 |
| Year of hospitalisation |  |  |  |  |
| 2016 | 0.95 | 0.83, | 1.08 | 0.412 |
| 2017 | 0.82 | 0.72, | 0.94 | 0.006 |
| 2018 | 0.73 | 0.63, | 0.84 | <0.001 |
| 2019 | 0.59 | 0.51, | 0.68 | <0.001 |
| 2020 | 0.41 | 0.36, | 0.48 | <0.001 |
| 2021 | 0.44 | 0.38, | 0.51 | <0.001 |
| 2022 | 0.18 | 0.15, | 0.21 | <0.001 |
| Socio-economic deprivation, SIMD |  |  |  |  |
| 2 | 0.88 | 0.80, | 0.98 | 0.017 |
| 3 | 0.97 | 0.85, | 1.10 | 0.608 |
| 4 | 0.98 | 0.85, | 1.13 | 0.769 |
| 5 | 0.99 | 0.84, | 1.17 | 0.916 |
| 6 | 1.18 | 0.99, | 1.42 | 0.069 |
| 7 | 0.88 | 0.73, | 1.06 | 0.179 |
| 8 | 0.62 | 0.51, | 0.77 | <0.001 |
| 9 | 0.90 | 0.74, | 1.10 | 0.293 |
| 10 (least deprived) | 0.61 | 0.48, | 0.77 | <0.001 |
